# Small bowel fistula with colorectal cancer and mesenteric lymph node metastasis: a report of two cases

**DOI:** 10.1093/jscr/rjad675

**Published:** 2023-12-30

**Authors:** Yasuhiro Ishiyama, Misato Ito, Sohei Akuta, Masatoshi Yoshizawa, Misuzu Yamato, Hiroto Tanaka, Takatsugu Fujii, Naoto Okazaki, Chikashi Hiranuma, Katsuya Deguchi, Yasumitsu Hirano

**Affiliations:** Department of Gastroenterological Surgery, Saitama Medical University International Medical Center, Hidaka, Japan; Department of Gastroenterological Surgery, Saitama Medical University International Medical Center, Hidaka, Japan; Department of Gastroenterological Surgery, Saitama Medical University International Medical Center, Hidaka, Japan; Department of Gastroenterological Surgery, Saitama Medical University International Medical Center, Hidaka, Japan; Department of Gastroenterological Surgery, Saitama Medical University International Medical Center, Hidaka, Japan; Department of Gastroenterological Surgery, Saitama Medical University International Medical Center, Hidaka, Japan; Department of Gastroenterological Surgery, Saitama Medical University International Medical Center, Hidaka, Japan; Department of Gastroenterological Surgery, Saitama Medical University International Medical Center, Hidaka, Japan; Department of Gastroenterological Surgery, Saitama Medical University International Medical Center, Hidaka, Japan; Department of Gastroenterological Surgery, Saitama Medical University International Medical Center, Hidaka, Japan; Department of Gastroenterological Surgery, Saitama Medical University International Medical Center, Hidaka, Japan

**Keywords:** colorectal cancer, multivisceral resection, small bowel mesenteric lymph node metastasis

## Abstract

A 65-year-old man presented to our hospital with complaints of diarrhea. Computed tomography showed a fistula with the small intestine, and a single incision laparoscopic low anterior resection for rectum with D3 dissection and partial resection of the small intestine were performed. Lymph node dissection, including a part of the inflow vessel area, was also performed because lymph node swelling was observed in the mesentery of the small intestine around the fistula. Histopathological analysis revealed that the lymph nodes in the small intestine were positive for metastasis. The patient was a 61-year-old woman who presented to our hospital with a chief complaint of diarrhea. A partial resection of the small intestine, including resection of the left hemicolectomy and lymph node dissection around the fistula, was performed at laparotomy. Histopathological examination revealed numerous lymph node metastases in the small intestinal mesentery.

## Introduction

Although it is recommended that colorectal cancer (CRC) be detected early and treated as soon as possible, it is not uncommon to detect obstructive CRC due to symptoms or CRC that has already invaded other organs [1].

Unfortunately, there have been only a few case reports of small bowel mesenteric lymph node metastasis in patients with CRC with small bowel invasion [2, 3], and the low frequency of small bowel mesenteric lymph node metastases makes it debatable whether systematic resection of small bowel lymph nodes should be performed in all patients with CRC with small bowel invasion.

In this report, we describe two cases of direct invasion of the small intestine by CRC and fistula formation, in which lymph node dissection of the mesentery of the small intestine was performed, and histopathological examination revealed metastasis in the lymph nodes of the small intestine.

## Case report

### Case 1

**Figure 1:**
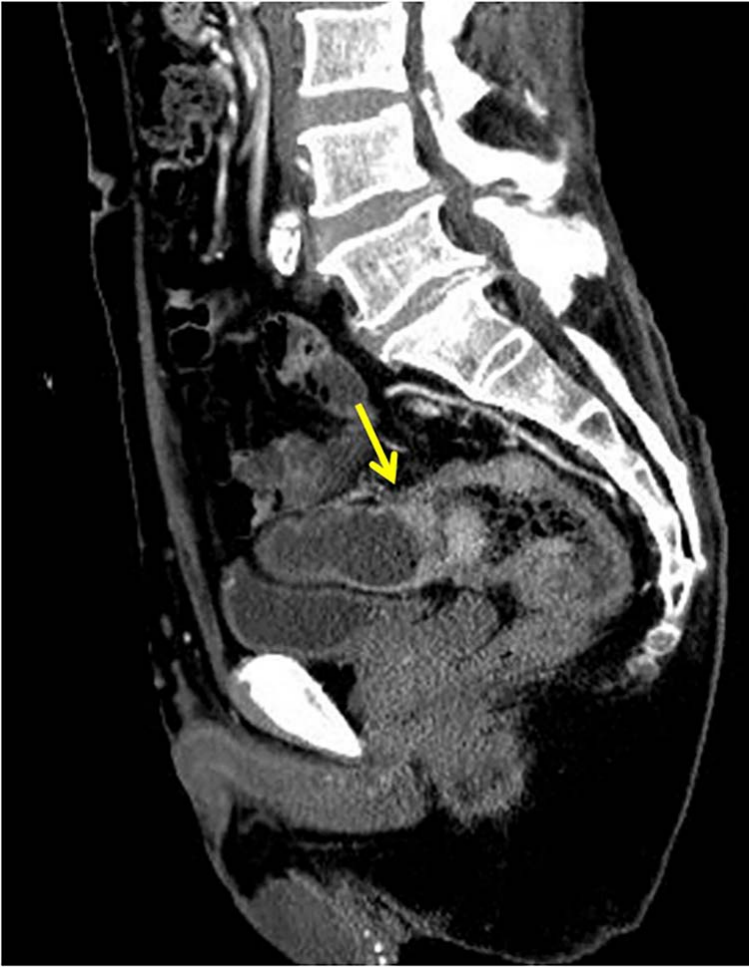
Fistula formation between the small intestine and the rectum was observed. Enlarged lymph nodes were also noted around the rectum.

**Figure 2:**
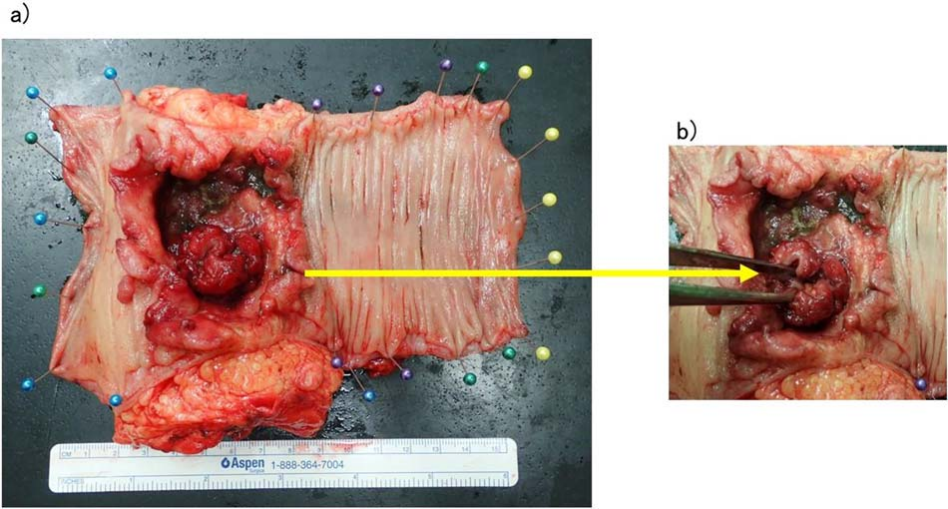
(a, b) Rectal cancer with small intestine invasion and fistula formation.

The patient was a 65-year-old man presenting to our hospital with complications of diarrhea for a year and weight loss. Colonoscopy showed rectal cancer on the anal verge (13 cm) with circumferential stenosis. Laboratory findings showed that the serum carcinoembryonic antigen (CEA) level was 4.7 μg/ml and that the serum carbohydrate antigen 19-9 (CA19-9) was 72.2 U/ml. A computed tomography (CT) scan of the abdomen showed thickening of the rectal wall with inflammation. Fistula formation between the ileum and the rectum was observed. Enlarged lymph nodes were also noted around the rectum and no enlarged mesenteric lymph nodes (Fig. 1). The patient was diagnosed with rectal cancer with ileum invasion and then scheduled to undergo surgery. We performed single-incision laparoscopic low anterior resection of the rectum with D3 dissection and partial resection of the ileum. Lymph node dissection, including the area of the inflow vessel, was also performed due to observed lymph node swelling in the mesentery of the ileum around the fistula formation intraoperatively. The operative time was 326 min, and the blood loss volume was 15 ml. Histopathological analysis revealed that the lymph nodes in the small intestine were positive for metastasis, and the diagnosis was pT4b (small intestine) N1 (2/16 (#251, 1/12, #252 1/4, #253 0/0)) M1 (small intestine mesenteric lymph node (1/1)), nonsolid type (por2) adenocarcinoma, ly3, v2 f Stage IV [TNM (tumor, nodes, metastasis) classification] (Fig. 2a and b). After discharge from the hospital, capecitabine plus oxaliplatin was administered for 6 months as adjuvant chemotherapy. The patient has been recurrence-free for 2 years after surgery.

### Case 2

**Figure 3:**
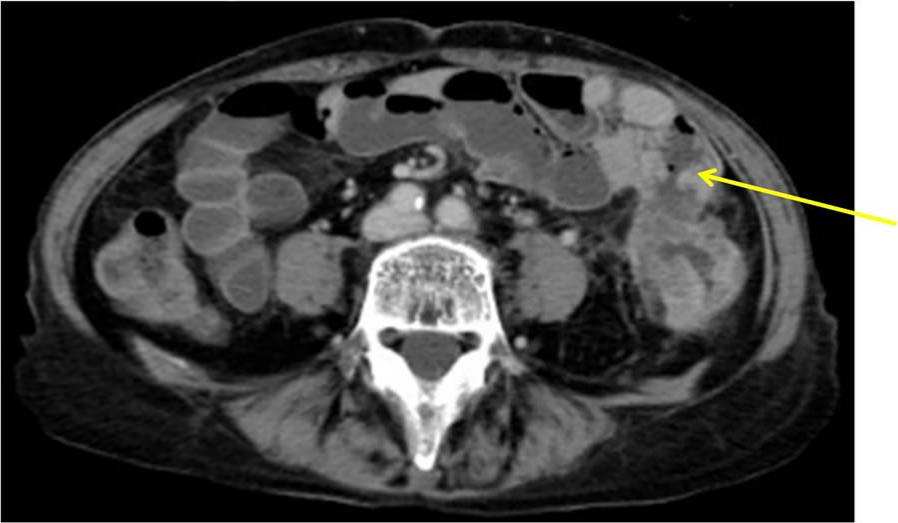
Descending colon with circumference tumor and small intestine invasion.

The patient was a 61-year-old woman presenting to our institution with the chief complaint of abdominal distension and diarrhea for a month. Colonoscopy showed descending colon cancer with all circumference-related stenosis. Her laboratory findings revealed a serum CEA level of 5.5 μg/ml and serum CA19-9 level of 778.4 U/ml. The CT scan results showed wall thickening in the descending colon. The descending colon on the proximal side of the tumor was dilated. The ileum is in contact with the tumor. Positron emission tomography (PET-CT) showed fluorodeoxyglucose (FDG) accumulation in the descending colon, mediastinal lymph nodes, enlarged mesenteric lymph nodes, and pararenal aortic lymph nodes (Fig. 3). The patient was preoperatively diagnosed as Stage IV, but she had obstructive symptoms, so surgery was planned. Laparotomy with left hemicolectomy and partial small bowel resection were performed. The ileum, including lymph node dissection around the fistula formation, was also resected.

**Figure 4:**
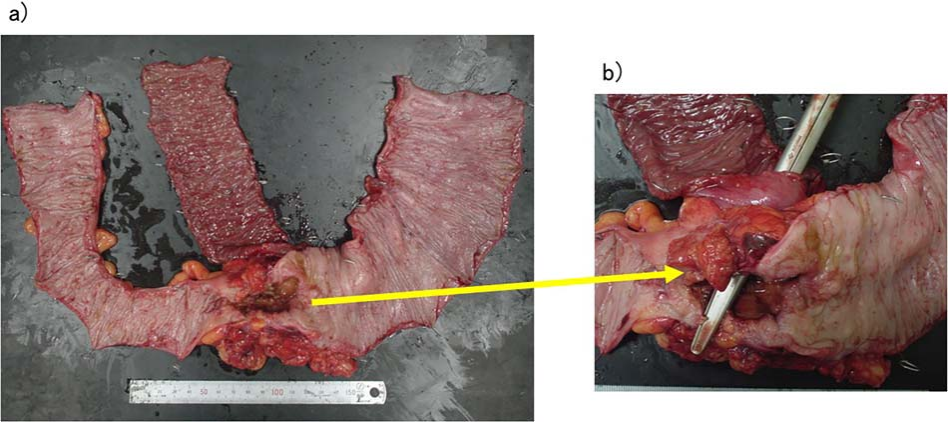
(a, b) Descending colon cancer with small intestine invasion and fistula formation.

The operative time was 234 min, and the blood loss volume was 155 ml. Histopathological analysis revealed numerous lymph node metastases within the mesentery of the small intestine, and the patient was diagnosed with pT4b (small intestinal mucosa) N3 (28/41) M1 (small mesenteric lymph node 6/8) f Stage IV (TNM classification), nonsolid type (por2) adenocarcinoma, ly3, v2 (Fig. 4a and b). After receiving chemotherapy, she died 18 months after surgery.

## Discussion

The frequency of fistula in colon cancer patients is very low. Welch *et al.* [4] reported that in only 13 among 2004 cases. The underlying mechanism of fistula formation is that adhesion to adjacent organs must occur prior to tumor invasion and that cancer must invade the adjacent organs within the adherent area. First, inflammation spreads around the colon due to destruction of the colonic wall structure by the cancer [5], and then a fistula can form due to the invasion of adjacent organs by the colon tumor and associated tissue necrosis [6].

On the other hand, the guidelines of the Japanese Society for Cancer of the Colon and Rectum recommend complete mesocolic excision with central vessel ligation for colon cancer [7]. However, the necessity of lymph node dissection for invasion of other organs, including invasion of the small intestine, is not mentioned, and considering the tumor status.

We examined 52 cases of CRC with suspected small intestinal invasion during the period from 2007 to 2019 at Saitama Medical University International Medical Center. In terms of invasion into the small intestine, 16 cases were found to have progressed deeper than the mucosal lamina propria, with 2 cases showing small intestinal mesenteric lymph node metastasis (Table 1).

**Table 1:** Characteristics of CRC with suspected small intestine invasion.

*n* = 52	
Age	70 (31-88)
Sex	
Male	28
Female	24
Tumor location	
Cecum	6
Ascending	6
Transverse	9
Descending	3
Sigmoid	19
Rectal	9
Surgical approach	
Laparoscopic	18
Conversion to open surgery	4
Open	34
Tumor differentiation	
Tub1	5
Tub2	31
Muc	4
Por	12
Sig	0
Lymphatic invasion	
+	18
-	34
Venous invasion	
+	36
-	16
Invasion depth of small intestine	
Mucosa	11
Submucosa	0
Mucosa propria	5
Serous membrane	4
No invasion	32

Histologically, 20 of the 52 cases showed invasion of the small intestine. In nine cases, tumor invasion was confined to the serosa in four cases and musclaris propria in five cases, and no lymph node metastasis to the small intestinal mesentery was observed in these cases. On the other hands, in two cases in which metastasis to lymph nodes in the mesentery of the small intestine was observed, both cases showed invasion deeper than the mucosal lamina propria and the formation of fistulas. These cases also showed lymph node metastasis to the mesentery at the primary site. In addition, combining the past literature and our own experience, six cases of small intestinal invasion of CRC with small mesenteric lymph node metastasis have been observed. Four of these cases had fistula formation in the small intestine (Table 2).

**Table 2:** Cases of small intestinal invasion of CRC with small mesenteric lymph node metastasis.

Case	Authors	Year	Sex	Age	Primary	Complain	Differentiation	Fistula	Invaded organ	Invasion depth of small intestine	Outcome
1	A Takiyama	2016	Woman	80	Sigmoid	Positive fecal occult blood test		−	Terminal ileum		Dead (31)
2	A Takiyama	2016	Man	79	Sigmoid	Constipation and weight loss		+	Jejunum		Dead (48)
3	A Takiyama	2016	Man	76	Ascending	Weight loss and appetite loss		−	Small bowel		Dead (67)
4	S Eto	2021	Man	67	Sigmoid	Weight loss and nausea	Tub2	+	Ieum	Submucosa	Alive ((21)
5	Our case	2022	Man	65	Rectal	Weight loss and diarrhea	Por2	+	Small bowel	Mucosa propria	Alive (24)
6	Our case	2022	Woman	61	Sigmoid	Diarrhea	Por2	+	Small bowel	Mucosa	Dead (18)

In conclusion, if lymph node metastases are suspected due to enlarged lymph nodes observed intraoperatively or on preoperative CT scans, lymph node dissection of the mesentery might be considered.

## Data Availability

The data that support the findings of this study are available on request from the corresponding author, [Y.I.]. The data are not publicly available due to [restrictions, e.g. their containing information that could compromise the privacy of research participants].
